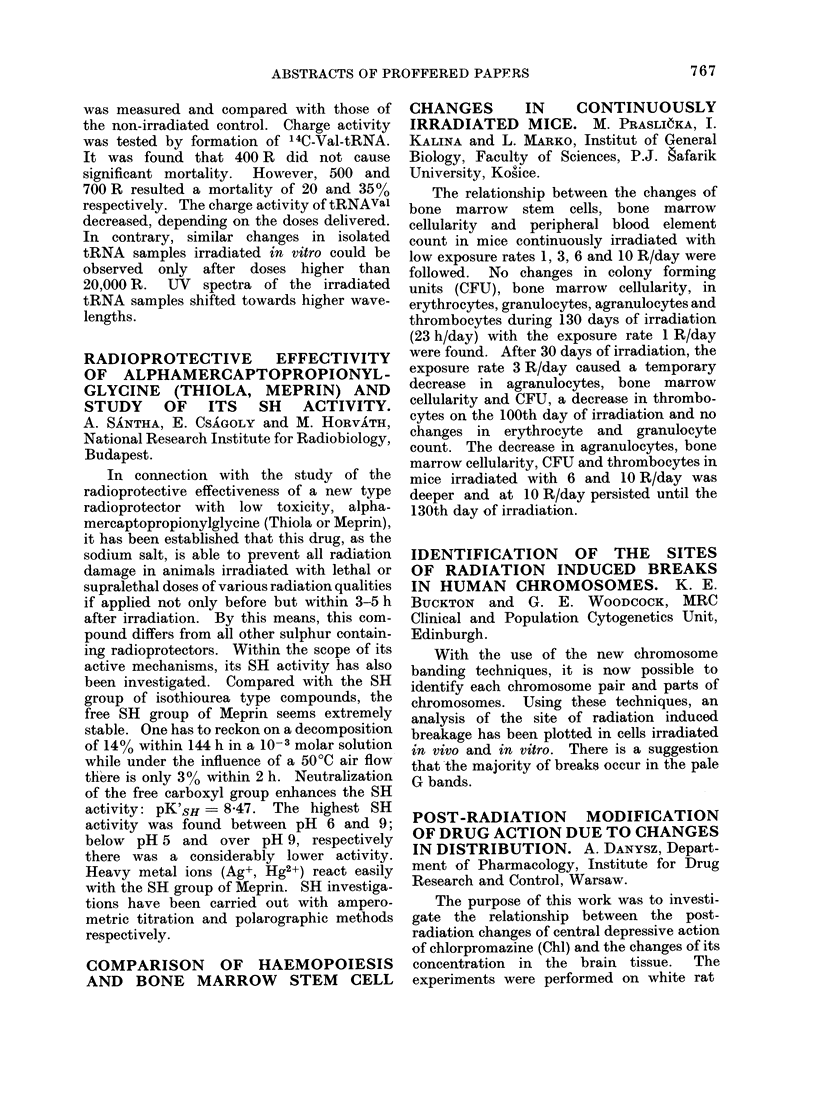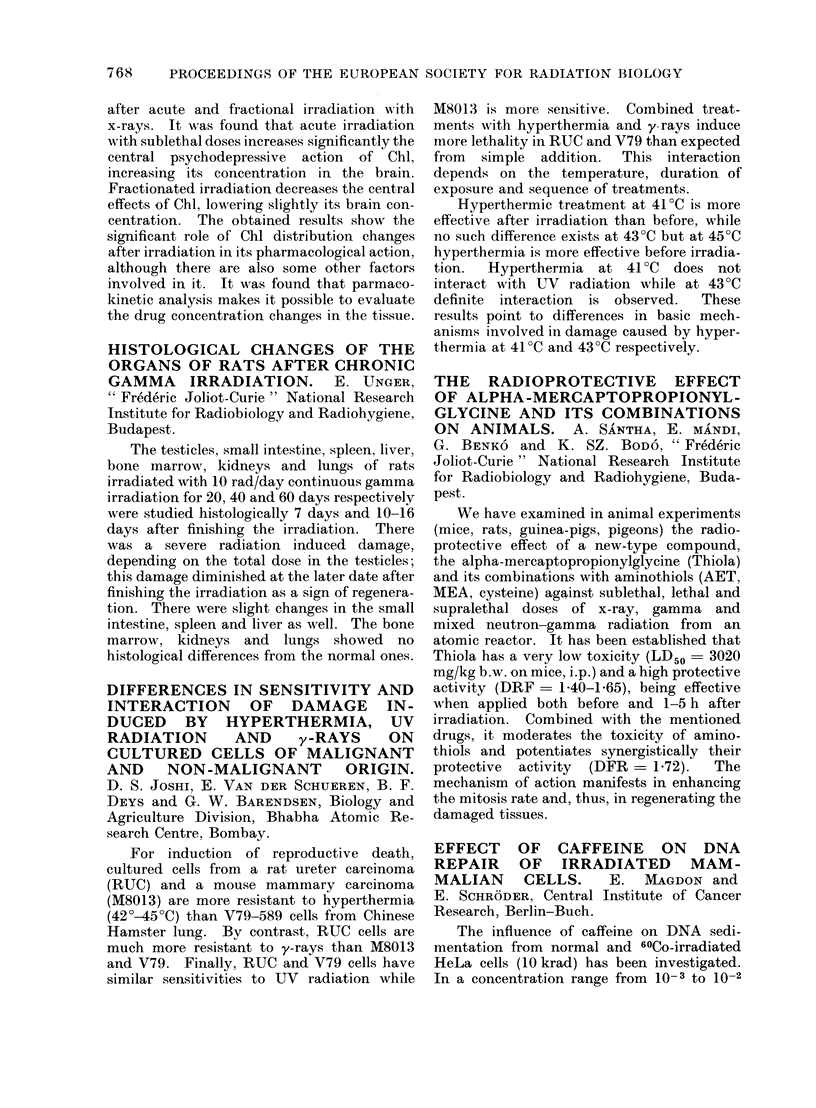# Proceedings: Post-radiation modification of drug action due to changes in distribution.

**DOI:** 10.1038/bjc.1975.344

**Published:** 1975-12

**Authors:** A. Danysz


### POST-RADIATION MODIFICATION OF DRUG ACTION DUE TO CHANGES IN DISTRIBUTION.

A. Danysz, Department of Pharmacology, Institute for Drug Research and Control, Warsaw.

The purpose of this work was to investigate the relationship between the post-radiation changes of central depressive action of chlorpromazine (Chl) and the changes of its concentration in the brain tissue. The experiments were performed on white rat after acute and fractional irradiation with x-rays. It was found that acute irradiation with sublethal doses increases significantly the central psychodepressive action of Chl, increasing its concentration in the brain. Fractionated irradiation decreases the central effects of Chl, lowering slightly its brain concentration. The obtained results show the significant role of Chl distribution changes after irradiation in its pharmacological action, although there are also some other factors involved in it. It was found that parmacokinetic analysis makes it possible to evaluate the drug concentration changes in the tissue.